# Globalization and health scholarship in a time of pandemic: from critical past to uncertain future

**DOI:** 10.1186/s12992-020-00563-6

**Published:** 2020-04-15

**Authors:** Ronald Labonté

**Affiliations:** School of Epidemiology and Public Health, Faculty of Medicine, University of Ottawa, 600 Peter Morand Crescent, Ottawa, Ontario K1G 5Z3 Canada

So many things in our lives became unhinged in a few short weeks: lock-downs, quarantines, social distancing, pandemic infection and death, an economic meltdown to rival 2008, and shortages in everything from medical supplies to food. Few nations or peoples in the world are unaffected, and how SARS-CoV2 and its COVID-2019 disease will eventually play out remains a riveting question mark for those of us with privileged lives, and an imminent threat for those without.

It is in this context that our journal finds itself celebrating its 15th year. Our publisher, BMC, has compiled a short list of our critical past, highlighting the most referenced articles from each of our years. The list tells us a story of how our journal has attempted to track the trajectory of globalization and its many health pathways, with embedded lessons for an uncertain future.

Unsurprisingly, our very first ‘big’ article (2005) did precisely that: it mapped the many ways in which health was already being, or could be, impacted by globalization [1]. This macro-look was matched by a more focused assessment of how unhealthy nutrition transitions and chronic disease rates (2006) were globalization’s fellow travellers [2]. A year later (2007), and as prelude to the work of Globalization Knowledge Network of the World Health Organization’s Commission on Social Determinants of Health, globalization was defined as a ‘determinant of social determinants’ trickling down in ways sometimes healthy, but predominantly health-risky [3].

The next two years featured more circumscribed top-end papers: the first on how ‘modernizing’ China (code for that country’s increased integration in the global economy) was experiencing a rapid transition to high rates of chronic disease (2008) [4]; and the second on how trade liberalization, one of globalization’s defining features, was doing the same in Central America (2009) [5]. The differential impacts on health of specific food items, in this case palm oil consumption, suggested that globalization’s health impacts were not equitably allocated (2011) [6]. Some of globalization’s unequal outcomes were manifest in health systems themselves: from radiology services (2010) [7], to biases that privilege research from rich countries over poorer ones (2017) [8], to this year’s so-far trending lead article on global surgery (2020) [9].

A recurrent journal theme over the years has also been development policy and health: initially pointing out how partnerships between richer (donor) and poorer (recipient) countries can be mutually beneficial (2012) [10], thus making the case for stronger international cooperation; and later lauding the bold, expansive directions of the Agenda 2030 and the Sustainable Development Goals (2015) [11]. The following year (2016) the necessary role of civil society to keep momentum moving captured readers’ interests [12].

In the wake of the 2008 financial crisis, we were also reminded of the importance of health investments in sustaining both human *and* economic health (2013) [13]. Economic health, however, is not necessarily good for human health. Three recent articles specifically cautioned us to be wary of globalization’s commercial drivers: from dominating online health information (2014) [14], to Big Tobacco’s game plans to sustain or expand its global markets (2018) [15], and the use of ‘front groups’ to advance corporate interests under a cloak of scientific neutrality (2019) [16].

Some of these leading article themes led to the creation of special journal collections. The large number of articles related to trade and health, for example, were recently gathered in a collection, with new articles being added as they are published [17]. Another collection focuses on health system innovations in low- and middle-income countries, belying the notion that such countries lack innovative ideas from which the world at large can learn [18]. The enduring topic of global health partnerships has its own dedicated place in the journal [19], while we recently hosted a special call for papers on intersectoral actions for health in the SDG that generated a range of new contributions [20]. There is also a new open collection, timely given the COVID-19 crisis, examining governance and preparedness for infectious disease threats [21]. The journal also published our first supplement in 2019 on the political determinants of health inequities and universal health coverage [22]. Many of these specially edited collections were published in collaboration with partnering universities and institutions.

Although such collections and the topics of our past 15 years of lead articles help to identify the parameters of our field of study, the journal has sometimes veered close to a more ‘international’ than ‘globalization and’ health perspective. A surge in single country health studies a few years ago had the editorial team reformulate more precisely the journal’s scope to ensure its focus on the importance of how globalization processes affected health outcomes, whether in a given country, cross-nationally interrogated, or examined at a global scale [23]. The accompanying re-design in our sections and editorial processes, and a revitalization of our Editorial Board, has made the scholarship we publish more germane to globalization and health researchers and, we hope, more useful to global health policy-makers and advocates.

Events since the start of this year, however, present the global health space with an oft-predicted but yet unpredictable pandemic challenge. We would be remiss if we didn’t then re-assess somewhat our journal’s future path. Although globalization (the increasing interdependence of peoples across time and space) is unlikely to cease, the economic rules that have largely defined and been driving it for the past 40 years are under ever-more critical questioning. The global supply chains that bound together our economies are under threat, nativist sentiments are fraying international cooperation, the (still under-regulated) world of financial speculation is once more haemorrhaging, and it is again the poor and the vulnerable who will bear the heaviest health burden.

How well our governments and our communitarian ethos (gun purchases or uber-elite retreats to secure boltholes notwithstanding) respond effectively to these new existential challenges is the order of the day. Climate change and environmental resource depletions may be taking a recessionary breather but wait around the corner. What new form(s) of globalization arise, and with what health equity outcomes, will be the necessary grist of our globalization and health scholarship of the near future.

## Data Availability

Not applicable
